# The Proangiogenic Effects of Melanoma-Derived Ectosomes Are Mediated by αvβ5 Integrin Rather than αvβ3 Integrin

**DOI:** 10.3390/cells13161336

**Published:** 2024-08-12

**Authors:** Magdalena Surman, Magdalena Wilczak, Małgorzata Bzowska, Grzegorz Tylko, Małgorzata Przybyło

**Affiliations:** 1 Department of Glycoconjugate Biochemistry, Institute of Zoology and Biomedical Research, Faculty of Biology, Jagiellonian University, 30-387 Krakow, Poland; magdalena.surman@uj.edu.pl (M.S.); magdalena.wilczak@doctoral.uj.edu.pl (M.W.); 2 Doctoral School of Exact and Natural Sciences, Jagiellonian University, 30-348 Krakow, Poland; 3 Department of Immunology, Faculty of Biochemistry, Biophysics and Biotechnology, Jagiellonian University, 30-387 Krakow, Poland; malgorzata.bzowska@uj.edu.pl; 4 Department of Cell Biology and Imaging, Institute of Zoology and Biomedical Research, Faculty of Biology, Jagiellonian University, 30-387 Krakow, Poland; grzegorz.tylko@uj.edu.pl

**Keywords:** angiogenesis, ectosomes, extracellular vesicles, integrins, melanoma

## Abstract

Ectosomes are carriers of proangiogenic factors during cancer progression. This study investigated whether the proangiogenic effect exerted by melanoma-derived ectosomes on recipient endothelial cells is mediated by ectosomal αvβ3 and αvβ5 integrins. Ectosomes were isolated from the conditioned culture media of four melanoma cell lines and melanocytes. Changes in gene and protein expression of αvβ3 and αvβ5 integrins, as well as VEGF and TNF-α were assessed in ectosome-treated endothelial cells. To confirm the functional involvement of ectosomal integrins in functional tests (Alamar Blue, wound healing and tube formation assays), ectosomes were also pretreated with anti-integrin antibodies and integrin-blocking peptides echistatin and cilengitide. Melanoma-derived ectosomes induced changes in the expression of αvβ3 and αvβ5 integrins in recipient endothelial cells, leading to increased viability, migratory properties, and tube formation potential. The extent of proangiogenic stimulation varied depending on the types of cells releasing ectosomes and the recipient cells. The use of anti-integrin antibodies and integrin-blocking peptides revealed a more significant role for the αvβ5 integrin/VEGF than the αvβ3 integrin/TNF-α pathway in the interactions between ectosomes and endothelial cells. The study demonstrated the functional role of ectosomal αvβ3 and αvβ5 integrins. It also provided a baseline understanding of ectosome-mediated αvβ3 integrin/TNF-α and αvβ5 integrin/VEGF signaling in angiogenesis.

## 1. Introduction

During carcinogenesis, the increasing size of a tumor requires an expansion of its blood vessel network. The initiation of angiogenesis involves the activation of various signaling pathways that lead to the proliferation and migration of vascular endothelial cells or their precursors. Over the years, the release of extracellular vesicles (EVs), ectosomes and exosomes has become recognized as one of the mechanisms that facilitate interactions between tumor and endothelial cells. EVs are small, lipid membrane-enclosed particles with a well-documented ability to transfer specific molecules between EV-releasing and recipient cells. Tumor-derived EVs can modulate essential biological processes in cancer cells, as well as functions of fibroblasts [1], endothelial cells [2], and/or immune cells [3]. Subsequent transfer of proangiogenic factors followed by changes in the structure of the extracellular matrix (ECM) indicate the important role of EVs in tumor angiogenesis.

There is growing evidence that tumor-derived ectosomes (EVs with a diameter in the range of 0.1–1 µm) either facilitate the transfer of several proangiogenic factors or enhance their expression in endothelial cells. To date, the functional effect on endothelial cell proliferation and migration has been demonstrated for ectosomes bearing vascular endothelial growth factor (VEGF) [4], matrix-degrading metalloproteinases (MMP-2 and MMP-9) and their endogenous activator CD147 [5], interleukin 6 (IL-6) [6], an oncogenic mutant of epidermal growth factor receptor (EGFRvIII) [7], sphingomyelin [8] and miR-1246 molecules [9]. The proangiogenic effect of ectosomes has also been observed in a mouse model. Mice inoculated with tumor-derived ectosomes showed greater endothelial cell mobilization and increased vascular density within tumor lesions [10].

In addition to growth factors, metalloproteinases, cytokines, miRNAs and lipids, ectosomes provide endothelial cells with integrins–transmembrane receptors directly involved in cell-matrix adhesion and with well-documented proangiogenic potential. Many integrins have already been detected in tumor-derived EVs; however, whether the cancer-promoting effect of EVs is mediated by integrins present in EV cargo remains an open question. The first study suggesting that EV-mediated transfer of integrins results in actual gain of function by recipient cells was conducted by Fedele et al. [11]. Exosomes released in vitro by stem-like prostate cancer (PrCa) cells were able to transfer αvβ6 integrin to tumor cells that did not express this receptor. Moreover, the exosomal transfer of αvβ6 integrin significantly altered the adhesion and migratory properties of recipient cells.

The largest body of research on the role of integrins in angiogenesis has focused on αvβ3 integrin, a vitronectin receptor that is often upregulated in developing blood vessels. Targeting αvβ3 integrin function with specific antibodies or its antagonists inhibited angiogenesis in various in vivo models, including murine melanoma [12]. Moreover, αvβ3 integrin-positive exosomes released by prostate cancer cells have been shown to be efficiently internalized by non-cancerous cells and led to increased αvβ3 integrin expression on the cell surface, as well as increased adhesion and migration of recipient cells [13]. This suggests that EVs may actively contribute to the αvβ3 integrin intercellular transfer.

Although αvβ3 integrin expression is abundant in endothelial cells undergoing angiogenesis, the involvement of αvβ3 integrin in angiogenic signaling pathways may not be general. Studies have demonstrated the presence of two cytokine-dependent angiogenic signaling pathways: (1) induced by tumor necrosis factor α (TNF-α) and requiring a functional αvβ3 integrin or (2) induced by VEGF and requiring a functional αvβ5 integrin [14]. So far, there have been no studies exploring which of the above-mentioned mechanisms is dominant for EV-associated integrins and whether the predominance of either αvβ3 integrin/TNF-α or αvβ5 integrin/VEGF pathway changes with tumor progression.

Therefore, the aim of this study was to investigate whether the proangiogenic effect exerted by melanoma-derived ectosomes on recipient endothelial cells is mediated by αvβ3 and αvβ5 integrins. We investigated the functional role of both integrins present in ectosomes derived from primary and metastatic melanoma cell lines, as well as non-transformed melanocytes, to provide a baseline understanding of ectosome-mediated αvβ3 integrin/TNF-α and/or αvβ5 integrin/VEGF signaling in tumor angiogenesis.

## 2. Materials and Methods

### 2.1. Antibodies and Other Reagents

RPMI 1640 GlutaMAX™-I medium, fetal bovine serum (FBS), MicroBCA Protein Assay kit, Alamar Blue cell viability reagent, Alexa Fluor™ 633 Phalloidin (cat. A22284), Geltrex™ LDEV-Free Reduced Growth Factor Basement Membrane Matrix and TaqMan™ Universal Master Mix II, with UNG were all purchased from Thermo Fisher Scientific (Waltham, MA, USA). Anti-CD63 mouse monoclonal primary antibody (clone RFAC4, cat. CBL553), anti-β-actin mouse monoclonal primary antibody (clone AC-15, cat. A1978), anti-ανβ3 integrin rabbit monoclonal primary antibody (clone EM22703, cat. ZRB1190), anti-ανβ5 integrin rabbit monoclonal primary antibody (clone EM09902, cat. ZRB1191), anti-VEGF-A mouse monoclonal primary antibody (clone 3F7, cat. SAB1402390), anti-TNF-α mouse monoclonal primary antibody (clone M1-C4, cat. SAB1404480), FITC-conjugated goat anti-rabbit IgG (H+L) secondary antibody (cat. 12-507), human VEGF-A ELISA Kit (cat. RAB0507), human TNF-α ELISA Kit (cat. RAB0476), Lumi-LightPLUS Western Blotting Kit (including anti-mouse and anti-rabbit IgG-HRP secondary antibodies), echistatin α1 isoform (cat. E2138), cilengitide trifluoroacetic acid salt (cat. SML1594), PKH67 Green Fluorescent Cell Linker Midi Kit for General Cell Membrane Labeling (cat. MIDI67), Fluoroshield™ mounting medium with DAPI, bovine serum albumin, trypsin-EDTA solution, and penicillin/streptomycin solution were obtained from Merck Group (Darmstadt, Germany). Mouse monoclonal primary antibodies for Arf6 (clone 3A-1, cat. sc-7971) and Hsp70 (clone C92F3A-5, cat. sc-66048) were purchased from Santa Cruz Biotechnology (Dallas, TX, USA). Melanocyte Growth Medium, Endothelial Cell Growth Medium, Endothelial Cell Growth Medium MV, and a Cell Detach Kit for primary cells were from PromoCell GmbH (Heidelberg, Germany). The 2× concentrated Laemmli Buffer was obtained from Bio-Rad Laboratories (Hercules, CA, USA). Total RNA Midi Kit for RNA isolation and TranScriba Kit for reverse transcription were obtained from A&A Biotechnology (Gdańsk, Poland). All remaining chemicals were of analytical grade and commercially available.

### 2.2. Cell Lines

Four human cutaneous melanoma cell lines obtained from the ESTDAB Melanoma Cell Bank (Tübingen, Germany) were used in the study. Isogenic WM115 (primary) and WM266-4 (metastatic) cell lines represented radial/vertical growth phase and lymph node metastasis, respectively [15]. The second pair was the primary melanoma WM793 cell line, representing the vertical growth phase [16], and WM1205Lu, a metastatic variant of the WM793 cell line [17]. Melanoma cells were maintained in RPMI 1640 medium with GlutaMAX-I, supplemented with 10% FBS, penicillin (100 unit/mL), and streptomycin (100 μg/mL).

As a reference, primary normal human epidermal melanocytes (NHEM cell line) (PromoCell GmbH, Heidelberg, Germany, cat. C-12400) isolated from the epidermis of juvenile foreskin were used. Melanocytes were maintained in a dedicated Melanocyte Growth Medium with a Supplement Mix, without antibiotics/antimycotics.

In addition, two endothelial cell lines, i.e., human umbilical vein endothelial cells (HUVECs, isolated from the vein of the umbilical cord of pooled donors, cat. C-12203) and primary human dermal microvascular endothelial cells (HDMECs, isolated from the dermis of juvenile foreskin, cat. C-12210) were purchased from Merck Group (Darmstadt, Germany). HUVECs and HDMECs were then maintained in a dedicated Endothelial Cell Growth Medium with a Supplement Mix, penicillin (100 unit/mL), and streptomycin (100 μg/mL).

All the aforementioned cell lines were cultured in monolayers in an atmosphere of 5% CO_2_ at 37 °C in a humidified incubator and passaged after reaching approximately 80% confluence.

### 2.3. Ectosome Isolation

Each ectosome sample was isolated from approximately 200 mL of conditioned media (~twenty 100 mm Petri dishes) from subconfluent (~80%) cultures of melanoma cells or melanocytes. Prior to isolation, subconfluent cells were maintained in serum-free media for 24 h. The conditioned media were then collected and subjected to sequential centrifugation. The remaining cells and cellular debris after centrifugations at 400× *g* (5 min, 4 °C) and 4500× *g* (20 min, 4 °C) were pelleted and discarded, while the supernatants were collected and concentrated using a low-vacuum filtration (LVF) procedure as described in [18]. LVF was performed on the dialysis membrane (Repligen, Waltham, MA, USA, cat. 131486) with 1000 kDa cut-off under low vacuum (−0.4 bar). Concentrated media (approx. 1.5–2 mL) were then centrifuged at 7000× *g* (20 min, 4 °C) to remove larger vesicles, followed by 18,000× *g* (20 min, 4 °C) to obtain ectosome pellets.

### 2.4. Assessment of Ectosome Sample Purity

The purity of ectosome samples was assessed by transmission electron microscopy (TEM) as in [19]. Vesicle size distribution and concentration were also evaluated by nanoparticle tracking analysis (NTA), as in [20]. Finally, Western blot analysis of EV markers was performed as previously described [20]. Briefly, whole-cell protein extracts and ectosome samples containing 30 μg of protein (according to the MicroBCA method) were mixed 1:1 with 2× concentrated Laemmli Buffer and separated by 10% SDS-PAGE under reducing conditions and then transferred to the PVDF membrane. After being blocked with 3% BSA in TBS/Tween, blots were probed with mouse monoclonal primary antibodies for CD63 (dilution 1:2000), Hsp70 (dilution 1:2000), and Arf6 (dilution 1:500) and then with goat anti-mouse secondary antibody (dilution 1:5000) conjugated to horseradish peroxidase (HRP). Bands were detected using HRP substrates from Lumi-Light PLUS Western Blotting Kit and ChemiDoc Imaging System (Bio-Rad, Hercules, CA, USA).

### 2.5. Incorporation of Ectosomes Derived from Melanoma Cell Lines and Melanocytes by Recipient Endothelial Cells

HUVEC or HDMEC cells (5 × 10^4^) were seeded on 12 mm microscope coverslips placed in 24-well plates and allowed to adhere for ~4 h. Isolated ectosomes were stained with a PHK67 kit according to the manufacturer’s instructions and added to HUVEC/HDMEC cells in the amount of 60 μg of protein (according to the MicroBCA method) for 18 h of incubation. The next day, endothelial cells were washed with PBS, fixed (4% formaldehyde in PBS, methanol-free), washed again and permeabilized (0.2% Tween in PBS). After another wash, 200 μL of Alexa Fluor™ 633 Phalloidin DMSO working solution (final concentration of 66 μM) was added to each slide for 1 h. After the final wash, the cover glass with stained cells was transferred onto the basic glass slide with a drop of Fluoroshield-DAPI mounting media. Control cells without the addition of ectosomes were also stained.

In addition, 5 × 10^4^ HUVEC/HDMEC cells were seeded into 24-well plates without cover glasses. Similarly, PHK67-stained ectosomes were added in the amount of 60 μg of protein (according to the MicroBCA method) for 18 h. Cells were then collected by trypsinization and analyzed with the use of a flow cytometer (BD FACS Calibur™ Flow Cytometer) in the green fluorescence channel (488 nm) for the percentage of positive cells (which incorporated ectosomes) and their relative fluorescence intensity. Moreover, after incubation with ectosomes, cells were washed and lysed for 15 min with 0.1% SDS-Tris buffer, and the fluorescence of the lysates was measured in a plate reader (490/502 nm). Control cells without the addition of ectosomes were also analyzed in both experiments.

### 2.6. Assessment of Total Protein Expression of αvβ3 and αvβ5 Integrins in Melanoma Cell Lines, Melanocytes and Ectosomes Derived from Them, and in Endothelial Cells after Incubation with Ectosomes

Integrin expression was analyzed by immunoblotting, analogous to that described in Section 2.4, using rabbit monoclonal primary antibodies anti-αvβ3 integrin (1:1000 dilution) and anti-αvβ5 integrin (1:2000) and goat anti-rabbit HRP-conjugated secondary antibody (1:5000). A mouse monoclonal anti-β-actin antibody (1:10,000) was used as loading control. Protein lysates of melanoma cells and ectosomes released by them, as well as protein lysates of HUVEC/HDMEC cells after 18 h of incubation with ectosomes at the amount of 60 µg of protein (determined after isolation using the Micro BCA method), were used for analyses. Control endothelial cells without the addition of ectosomes were also analyzed.

### 2.7. Assessment of Surface Expression of αvβ3 and αvβ5 Integrins in Endothelial Cells after Incubation with Ectosomes

Equal amounts of 5 × 10^4^ HUVEC or HDMEC cells were seeded into 24-well plates and allowed to adhere for ~4 h. Then, isolated ectosomes were added in the amount of 60 μg of protein (according to the MicroBCA method) for 18 h. After incubation, ectosome-treated and control cells were harvested by trypsinization, washed with PBS, and counted, and 5 × 10^4^ cells were stained with 0.1 μg (in 50 μL of PBS with 0.5% BSA) of rabbit monoclonal anti-αvβ3integrin or anti-αvβ5 integrin primary antibodies for 60 min at 4 °C. After washing with cold PBS, cells were incubated with secondary FITC-conjugated goat anti-rabbit IgG for 30 min at 4 °C. After another wash, all samples (unstained controls, secondary controls and fully stained endothelial cells) were analyzed by flow cytometry using LSRII cytometer (Becton Dickinson, USA). Forward and side scatter signals were used to gate for endothelial cells, and 10^4^ cells were acquired. The analysis was performed using BD FACSDiva software to determine the percentage and mean fluorescence intensity of positive cells. Histogram overlays were created using FCS Express™ RUO release: 7.22.0031 (De Novo Software, Pasadena, CA, USA).

### 2.8. Analysis of Gene Expression for αv, β3 and β5 Integrin Subunits in Endothelial Cells after Incubation with Ectosomes

Equal amounts of 5 × 10^4^ HUVEC or HDMEC cells were seeded in 24-well plates and allowed to adhere for ~4 h. Then, isolated ectosomes were added in the amount of 60 μg of protein (according to the MicroBCA method) for 18 h. After incubation, gene expression of integrin subunits was analyzed using RT-qPCR. Total RNA was isolated from control and ectosome-treated endothelial cells using Total RNA Midi Kit according to the manufacturer’s instructions. Five μg of RNA from each isolated sample was then reverse-transcribed using the TranScriba Kit, including 50 mM oligo(dT)_18_ primer according to the manufacturer’s instruction. The concentration of cDNA was assessed using a NanoDrop 2000 spectrophotometer, and 250 ng was taken for single reactions. Gene expression assays were performed using TaqMan^®^ Universal Master Mix II, no UNG (Life Technologies), according to the manufacturer’s protocol. Real-time PCR reactions were performed for 40 cycles of denaturation (15 s, 95 °C), annealing and elongation (1 min, 60 °C) using the StepOne Plus system (Applied Biosystems, Waltham, MA, USA). Housekeeping (*YWHAZ*) and target (*ITGAV, ITGB3*, *ITGB5*) gene-specific mRNAs were amplified with the use of TaqMan™ Gene Expression Assays, as listed in Table 1. All reactions were performed with three biological and three technical replicates. The reaction results were analyzed using StepOne Software ver. 2.0 using the 2^−ΔΔCt^ method.

### 2.9. Analysis of Endothelial Cell Viability after Incubation with Ectosomes

Isolated ectosomes were pre-incubated for 1 h with anti-αvβ3 and anti-αvβ5 integrin antibodies (0.1 μg of antibody per 60 μg of ectosomal protein according to the MicroBCA method) or arginylglycylaspartic acid (RGD) peptide mimetics–cilengitide and echistatin (both 1 μM per 60 μg of ectosomal protein). The concentrations of anti-integrin antibodies and RDG mimetics used were based on [21,22], respectively. Equal amounts of 5 × 10^4^ HUVEC or HDMEC cells were seeded into 96-well plates and allowed to adhere for ~4 h. The FBS-containing medium was then changed to 100 μL of FBS-free medium, and endothelial cells were incubated with untreated or preincubated ectosomes added in the amount of 60 μg of protein (according to the MicroBCA method) for 18 h. After incubation, cell viability analysis was performed using the Alamar Blue assay. To this end, 10% of Alamar Blue reagent was added to each well, and after 2 h, fluorescence intensity was measured at 560/595 nm in a multi-well plate reader. Results were standardized to the untreated control (taken as 1).

### 2.10. Analysis of Migratory Properties Endothelial Cells after Incubation with Ectosomes

Isolated ectosomes were pre-incubated for 1 h with anti-αvβ3 and anti-αvβ5 integrin antibodies (0.1 μg of antibody per 60 μg of ectosomal protein according to MicroBCA method) or RGD mimetics–cilengitide and echistatin (both 1 μM per 60 μg of ectosomal protein). The concentrations of anti-integrin antibodies and RDG mimetics used were based on [21,22], respectively. HUVEC or HDMEC cells were cultured to confluence on six-well plates. The cell-coated surface was then scraped with a 200 µL pipette tip, and untreated or preincubated ectosomes were added in the amount of 60 μg of protein (according to the MicroBCA method). Wounds were left to heal for 18 h. Each wound was photographed in 10 separate fields immediately after scraping (0 h) and after 18 h. The average wound closure rate was assessed by multiple measurements of the wound diameter using Zeiss AxioVision Rel.4.8 image analysis software and calculated as follows:

$$wound\;closure\;rate=\frac{initial\;wound\;width\;\left(0\;h\right)-wound\;width\;after\;18\;h}{initial\;wound\;width}$$

Results were standardized to the untreated control (taken as 1).

### 2.11. Analysis of Tube-Formation Potential of Endothelial Cells after Incubation with Ectosomes

Isolated ectosomes were pre-incubated for 1 h with anti-αvβ3 and anti-αvβ5 integrin antibodies (0.1 μg of antibody per 60 μg of ectosomal protein according to the MicroBCA method) or RGD mimetics–cilengitide and echistatin (both 1 μM per 60 μg of ectosomal protein). The concentrations of anti-integrin antibodies and RDG mimetics used were based on [21,22], respectively. Wells of 24-well plates were covered with 100 μL of cooled (4 °C) Geltrex™ LDEV-Free Reduced Growth Factor Basement Membrane Matrix. After polymerization of the matrix (30 min, 37 °C), equal amounts of 5 × 10^4^ HUVEC or HDMEC cells were seeded and allowed to adhere for ~4 h. Then, untreated or preincubated ectosomes were added in the amount of 60 μg of protein (according to the MicroBCA method). After 18 h, multiple images of each well were taken, binarized, and analyzed using the Angiogenesis Analyzer plug-in for ImageJ software (National Institutes of Health, Bethesda, MD, USA). The parameters considered included total tube length, number of closed tubes, and number of branches and junctions.

### 2.12. Analysis of TNF-α and VEGF Gene and Protein Expression, and Their Secretion by Endothelial Cells after Incubation with Ectosomes

Expression of *TNFA* and *VEGFA* genes and proteins in HUVEC and HDMEC cells after incubation with ectosomes was analyzed by RT-qPCR and immunoblotting, respectively, analogously as described in Section 2.4 and Section 2.8. For RT-qPCR, mRNAs specific for housekeeping (*YWHAZ*) and target (*TNFA* and *VEGFA*) genes were amplified using TaqMan™ Gene Expression Assays, as listed in Table 1. For immunoblotting, primary mouse monoclonal mouse anti-TNF-α (1:500 dilution) and anti-VEGF-A (1:1000) antibodies were used, as well as goat-anti mouse HRP-conjugated secondary antibody (1:5000).

### 2.13. Statistical Analysis

Unless stated otherwise, experiments were performed in three biological replicates. Analyses of variance (one-way ANOVA) and post-hoc Tukey’s tests were performed with the use of Statistica 13.3 software to test for statistically significant differences with *p* < 0.05.

## 3. Results

### 3.1. Assessment of Ectosome Sample Purity

**Figure 1:**
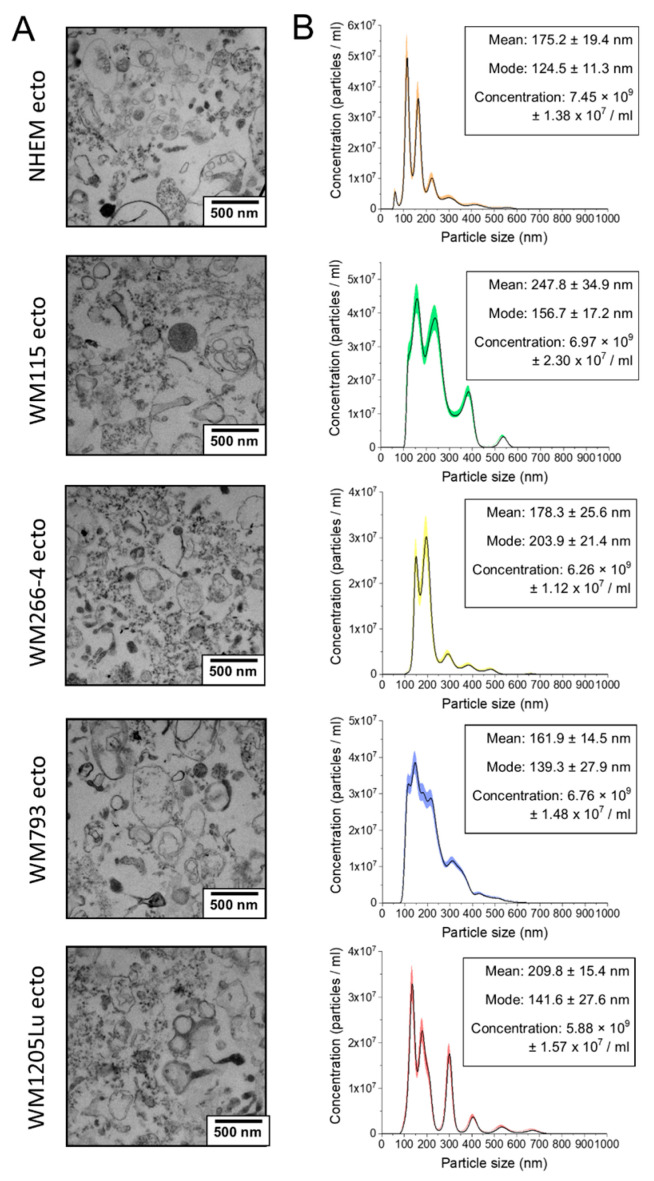
Analysis of purity and size distribution of melanocyte- and melanoma-derived ectosomes. (**A**) TEM imaging. (**B**) Nanoparticle Tracking Analysis (NTA). The means of five independent measurements (black lines) are presented on histograms. The colored area depicts ± standard deviation. Ectosomes were derived from NHEM—normal human epidermal melanocytes; WM115 (primary) and WM266-4 (metastatic)—melanoma cell lines originating from the same individual, radial/vertical growth phase and lymph node metastasis, respectively; primary WM793 cell line—representing the vertical growth phase; WM1205Lu cells—a metastatic variant of WM793 cells obtained from lung metastasis.

In this study, ectosomes were isolated from conditioned culture media of four human cutaneous melanoma cell lines (WM115, WM266-4, WM793, and WM1205Lu) and from normal human epidermal melanocytes (NHEM). A methodology involving LVF and subsequent centrifugation at 18,000× *g* was employed for isolation. According to the guidelines outlined by the International Society for Extracellular Vesicles (ISEV), ectosome samples were analyzed for particle size, concentration, and expression of EV protein markers. TEM examination (Figure 1A) confirmed the absence of cellular contaminants, organelles, or other debris, instead revealing heterogeneous populations of intact vesicles. Size distribution profiles were obtained using NTA (Figure 1B), demonstrating that the majority of vesicles fell within the typical size range of ectosomes (i.e., 100–1000 nm) with predominant subpopulations observed between 100–400 nm.

**Figure 2:**
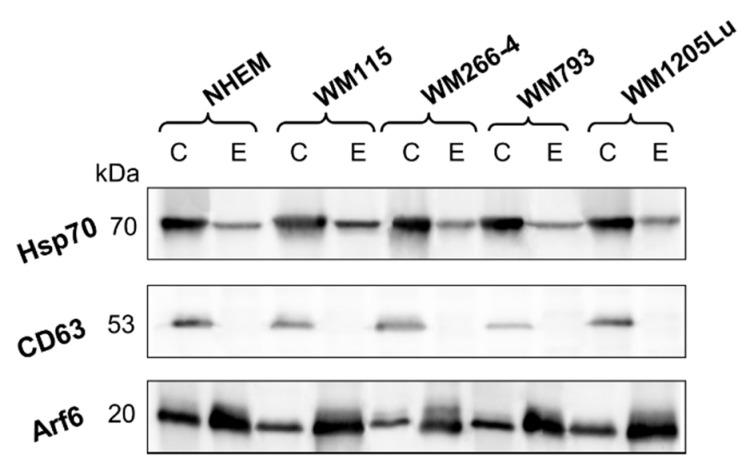
Western blot analysis of extracellular vesicle markers. Thirty micrograms of proteins from whole-cell protein extracts (lines C) and ectosome samples (lines E) separated by 10% SDS-PAGE and transferred to the PVDF membrane were probed with anti-CD63, anti-Hsp70, and anti-Arf6 as primary antibodies and anti-mouse IgG-HRP as a secondary antibody.

Finally, the expression of specific EV protein markers, namely Hsp70 and CD63 tetraspanin (considered exosomal markers), along with Arf6 (an ectosomal marker), was evaluated (Figure 2). A comparative analysis revealed a reduction in Hsp70 and lack of CD63 expression in the isolated ectosome samples when compared to reference whole cell lysates. On the contrary, there was an augmentation in the expression of Arf6, a protein intricately involved in ectosomes biogenesis. The characterized EV populations can be identified as ectosome-enriched and exosome-depleted.

### 3.2. Incorporation of Melanoma- and Melanocyte-Derived Fluorescently Stained Ectosomes by Recipient Endothelial Cells

**Figure 3:**
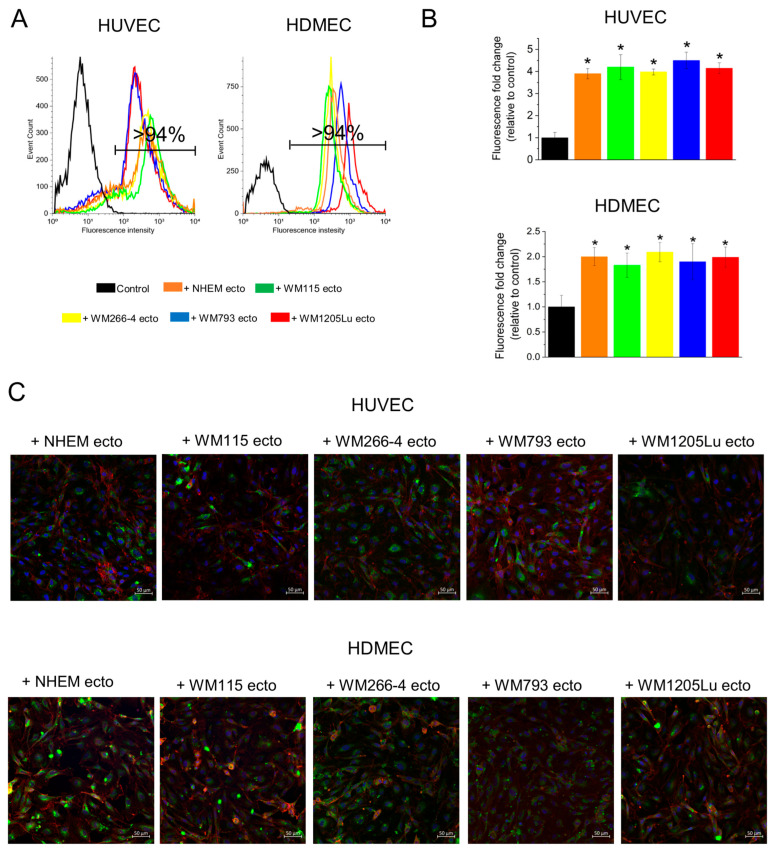
Analysis of the incorporation of melanoma- and melanocyte-derived ectosomes by recipient endothelial cells. Ectosomes were labeled with the fluorescent dye PKH67. Following 18 h of coincubation with ectosomes, endothelial cell fluorescence was analyzed using a flow cytometer, multi-well plate reader and confocal microscope. (**A**) Representative histograms from flow cytometry analysis. (**B**) Endothelial cell lysate fluorescence was measured in a multi-well plate reader (490/502 nm). (**C**) Confocal microscope imaging of the incorporation of melanoma- and melanocyte-derived ectosomes by recipient endothelial cells. Isolated ectosomes were labeled with the green fluorescent dye PKH67. Following an 18 h incubation period with ectosomes, recipient endothelial HUVEC and HDMEC cells were subsequently stained with Alexa Fluor™ 633 Phalloidin to visualize the cytoskeleton (red) and DAPI to visualize the nucleus (blue). All experiments were performed in triplicate. “*” indicates statistically significant differences compared to the control (Tukey’s post-hoc test, *p* < 0.05).

To evaluate the extent of ectosome uptake by HUVEC and HDMEC endothelial cells, ectosomes were labeled with the fluorescent dye PKH67. Following 18 h of coincubation with the labeled ectosomes, endothelial cell fluorescence was analyzed using a flow cytometer and quantified with a multi-well plate reader. Flow cytometry analysis (Figure 3A) demonstrated that >94% of endothelial cells treated with ectosomes exhibited fluorescence derived from PKH67. Moreover, when HUVECs were assessed using a multi-well plate reader, a substantial elevation of lysate fluorescence (3.9- to 4.5-fold) was observed after incubation with ectosomes derived from all cell lines relative to untreated HUVEC cells (Figure 3B). Conversely, HDMEC cells displayed a comparatively lower increase in fluorescence (1.8 or 2.1-fold) (Figure 3B).

Furthermore, confirmation of ectosome uptake was achieved by confocal microscopy imaging, as depicted in Figure 3C. In endothelial cells treated with ectosomes, the fluorescent signal from the internalized ectosomes exhibited a distinct localization pattern, predominantly surrounding the nucleus.

### 3.3. Analysis of αvβ3 and αvβ5 Integrin Protein Expression Levels in Melanoma Cells, Melanocytes, and Their Released Ectosomes

**Figure 4:**
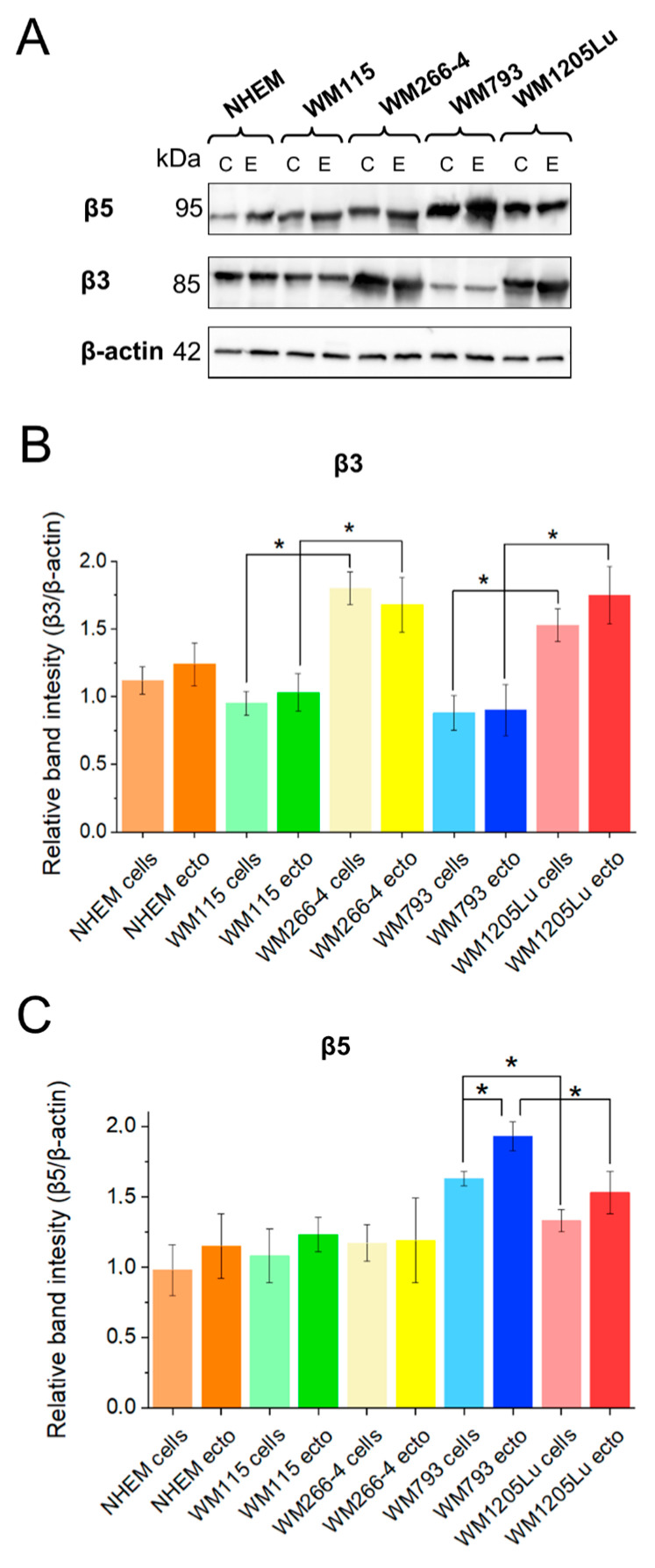
Western blot analysis of total αvβ3 and αvβ5 integrin expression in melanocytes and melanoma cell lines, and the corresponding derivative ectosome samples. Thirty micrograms of proteins from whole-cell protein extracts (lines C) and ectosome samples (lines E) were separated by 10% SDS-PAGE and transferred to the PVDF membrane. Membranes were then probed with rabbit monoclonal primary antibodies anti-αvβ3 (1:1000 dilution) and anti-αvβ5 (1:2000), and goat anti-rabbit HRP-conjugated secondary antibody (1:5000). Mouse monoclonal anti-β-actin antibody (1:10,000) was used as loading control. (**A**) Representative Western blots. (**B**,**C**) Densitometric analysis of αvβ3 and αvβ5 integrin expression relative to β-actin. All experiments were performed in triplicate. “*” indicates statistically significant differences (Tukey’s post-hoc test, *p* < 0.05).

The functional effect of αvβ3 and αvβ5 integrin-bearing ectosomes may depend on the abundance of these integrins within ectosomes. Therefore, the total protein expression levels of both cell adhesion receptors in ectosomes and their parental melanoma cells/melanocytes were assessed using immunoblotting techniques (Figure 4A). Metastatic WM1205Lu cells and their derived ectosomes exhibited elevated expression levels of αvβ3 integrin compared to the isogenic primary WM793 cells and their respective ectosomes (Figure 4B). This trend was similarly observed in another isogenic pair, namely WM115 (primary) and WM266-4 (metastatic) cells and their corresponding ectosomes. These findings align with previous studies indicating a positive correlation between αvβ3 integrin expression and melanoma progression, with β3 subunit expression being associated with the occurrence of lung metastases [23] (notably, the WM1205Lu cell line was derived from lung metastasis). In contrast, the expression of αvβ3 integrin in melanocytes was at a similar level as in both primary melanoma cell lines, i.e., WM115 and WM793. Furthermore, αvβ3 integrin expression levels remained consistent between the cells and their derived ectosome samples.

In advanced melanomas, a characteristic loss of αvβ5 integrin expression is commonly noted [24,25], impacting the adhesion properties of metastatic cells. Notably, cells and ectosomes originating from the metastatic WM1205Lu cell line exhibited diminished expression of this receptor in comparison to cells and ectosomes derived from the primary cell line WM793 (Figure 4C). In addition, ectosomes derived from the WM793 line demonstrated higher αvβ5 integrin expression levels than the cells from which they were released. Similar differences were not observed in the case of WM115 and WM266-4 cells and their respective ectosomes, where the expression levels remained similar to those observed in normal melanocytes.

### 3.4. Changes in αvβ3 and αvβ5 Integrin Protein and Gene Expression in Endothelial Cells Following Incubation with Melanoma/Melanocyte-Derived Ectosomes

**Figure 5:**
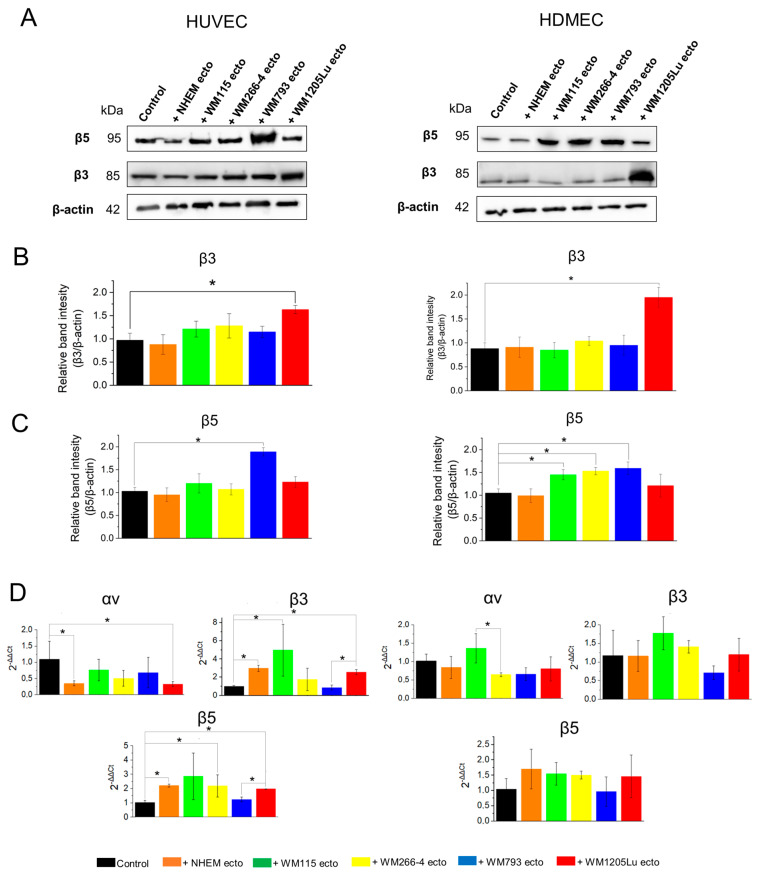
Analysis of αvβ3 and αvβ5 integrin total protein and gene expression in endothelial cells after 18-h of incubation with melanocyte- and melanoma-derived ectosomes. (**A**) Representative Western blots. Thirty micrograms of proteins from whole-cell protein extracts were separated by 10% SDS-PAGE and transferred to the PVDF membrane. Membranes were then probed with rabbit monoclonal primary antibodies anti-αvβ3 (and anti-αvβ5), and goat anti-rabbit HRP-conjugated secondary antibody. Mouse monoclonal anti-β-actin antibody was used as a loading control. (**B**,**C**) Densitometric analysis of αvβ3 and αvβ5 expression relative to β-actin. (**D**) RT-qPCR analysis of gene expression for αv, β3 and β5 integrin subunits in endothelial HUVEC and HDMEC cells after 18 h incubation with melanocyte- and melanoma-derived ectosomes. Housekeeping (*YWHZ*) and target (*ITGAV*, *ITGB3*, *ITGB5*) gene-specific mRNAs were amplified from 250 ng of cDNA with the use of TaqMan™ Gene Expression Assays. Analysis of relative gene expression was performed using the 2^−ΔΔCt^ method. All experiments were performed in triplicate. “*” denotes statistically significant differences (Tukey’s post-hoc test, *p* < 0.05).

In the next step, alterations in both protein and gene expression of αvβ3 and αvβ5 integrins in endothelial cells were evaluated after an 18 h incubation period with ectosomes. Notably, an elevation in total αvβ3 integrin protein expression was observed in both HUVECs and HDMECs exclusively after incubation with ectosomes derived from WM1205Lu cells (Figure 5A–C). Conversely, regarding αvβ5 integrin, an increase in expression was only discerned in HUVEC cells subsequent to incubation with ectosomes originating from WM793 cells, which aligns with the notably higher expression levels of αvβ5 integrin observed in these ectosomes. In HDMEC cells, augmented expression of αvβ5 integrin was evident following incubation with ectosomes derived from WM793 cells, as well as those derived from WM115 and WM266-4 cells.

The RT-qPCR technique was employed to evaluate the aforementioned changes in gene expression for integrin subunits (Figure 5D). In HUVECs, the notable increase in gene expression for the β3 subunit was observed subsequent to incubation with ectosomes derived from melanocytes (3-fold increase), WM115 melanoma cells (4.5-fold increase) and WM1205Lu (2.5-fold increase), as illustrated in Figure 5D. Similarly, gene expression for the β5 subunit in HUVECs exhibited augmentation following incubation with ectosomes derived from melanocytes, as well as WM115, WM266-4 and WM1205Lu cells, with fold increases of 1.8, 2.7, 1.9 and 2, respectively. Conversely, no discernible alterations in the expression of the αv subunit were noted in ectosome-treated HUVEC cells. In contrast, for HDMEC cells, no significant changes in gene expression were observed for any of the integrin subunits tested (Figure 5D). Interestingly, ectosomes derived from isogenic WM793 and WM1205Lu cells showed significant differences in their effect on the expression of β3 and β5 integrin subunit genes in HUVEC cells. Ectosomes derived from metastatic WM1205Lu cells caused a higher increase than their primary counterparts. Similar observations were not made for HDMEC cells. However, for this endothelial cell line, stimulation of αν subunit gene expression was significantly weaker for ectosomes derived from metastatic WM266-4 cells than from isogenic primary WM115 cells.

**Figure 6:**
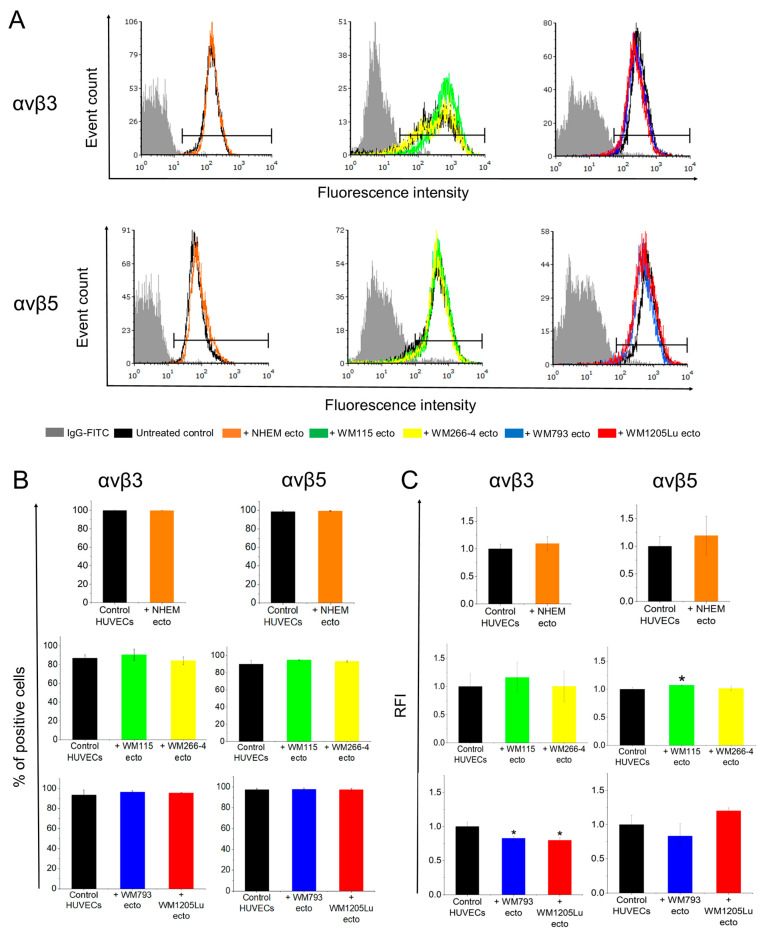
Flow cytometry analysis of αvβ3 and αvβ5 integrin surface expression in HUVEC cells following 18 h of incubation with melanocyte- and melanoma-derived ectosomes. After the incubation, 5 × 10^4^ cells were collected, indirectly labeled with rabbit monoclonal primary anti-αvβ3 integrin antibody and secondary FITC-conjugated goat anti-rabbit IgG, and subsequently analyzed by flow cytometry. (**A**) Representative histograms are depicted, where the gray-shaded histograms represent background signals acquired from secondary antibody staining. Based on these signals, the histogram markers were set, delineating the histogram sections corresponding to αvβ3 integrin- or αvβ5 integrin-positive HUVEC cells. (**B**) Surface expression of αvβ3 integrin on HUVEC cells presented as the percentage of positive cells and (**C**) relative fluorescence intensity of specific staining. All experiments were performed in triplicate. “*” indicates statistically significant differences compared to the control (Tukey’s post-hoc test, *p* < 0.05).

Finally, flow cytometry was utilized to examine the impact of ectosomal transfer on the surface expression of integrins in endothelial cells, considering that integrins are membrane receptors (Figure 6, Supplementary Figures S1 and S2). In both untreated and ectosome-treated HUVECs and HDMECs, a substantial percentage of αvβ3 integrin- and αvβ5 integrin-positive cells was observed, ranging between 80.7 and 99.7%, with minimal statistical variances between compared groups. However, noticeable differences were observed in the relative fluorescence intensity (RFI) values, which reflect the abundance of a specific antigen on the cell surface. Specifically, the fluorescence intensity of αvβ5 integrin-positive HUVEC cells exhibited an increase following incubation with ectosomes derived from WM115 cells. Conversely, the fluorescence intensity of αvβ3 integrin-positive HUVECs decreased after exposure to ectosomes derived from WM793 and WM1205Lu cells (Figure 6). Notably, ectosomes did not induce any changes in the surface expression of integrins in HDMEC cells (Supplementary Figures S1 and S2).

### 3.5. Functional Impact of Melanoma/Melanocyte-Derived Ectosomes Carrying αvβ3 and αvβ5 Integrins on Endothelial Cells Viability and Migration

**Figure 7:**
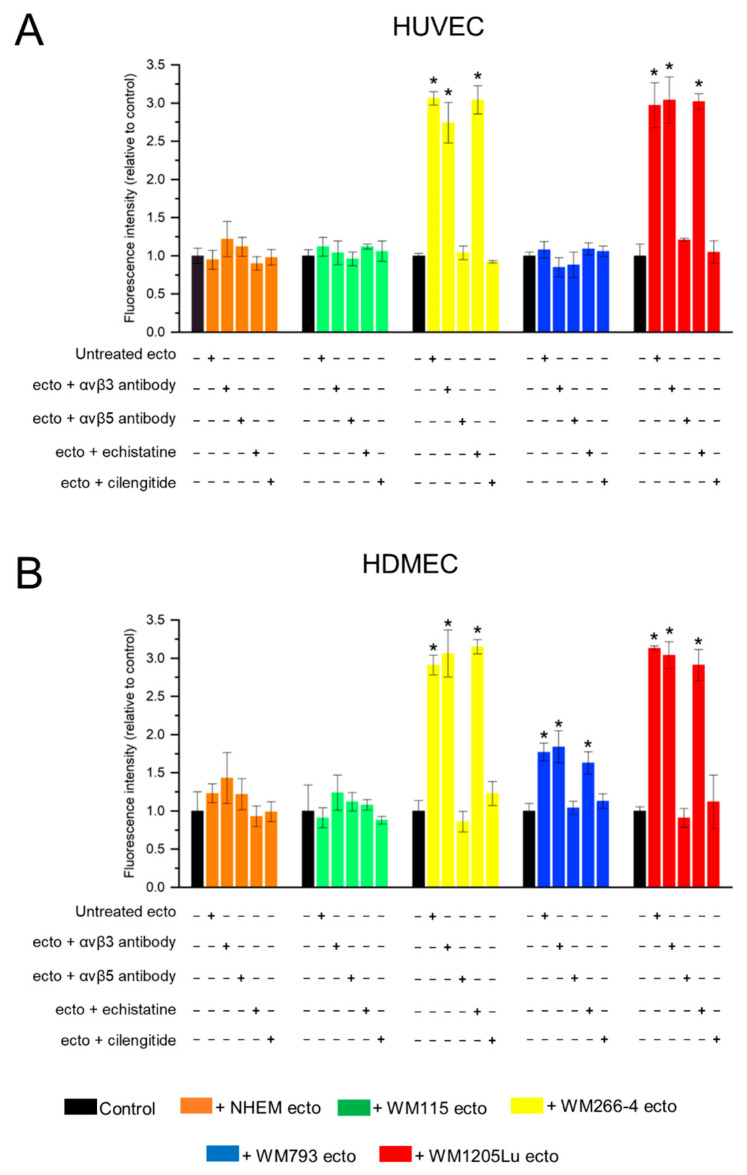
Alamar Blue cell viability assay performed on endothelial cells after 18 h of incubation with ectosomes derived from melanocytes and melanoma cells. The isolated ectosomes were pre-incubated with anti-αvβ3 and anti-αvβ5 integrin antibodies or the RGD mimetics (cilengitide and echistatin) and then added to 5 × 10^4^ of HUVEC (**A**) or HDMEC (**B**) cells. After 18 h of incubation with ectosomes, Alamar Blue reagent was added to each well and fluorescence intensity was measured at 560/595 nm in a multi-well plate reader. Results were normalized against the untreated control. All experiments were performed in triplicate. “*” indicates statistically significant differences compared to the control (Tukey’s post-hoc test, *p* < 0.05).

The subsequent investigation aimed to assess the influence of melanoma/melanocyte-derived ectosomes on endothelial cells’ viability and migratory capabilities. Specifically, the extent to which the proangiogenic effects of ectosomes are attributed to the presence of αvβ3 and αvβ5 integrins was examined using specific antibodies and RGD mimetics to block integrins, i.e., echistatin (αvβ3 integrin inhibitor) and cilengitide (αvβ3 and αvβ5 integrin inhibitor), in addition to untreated controls. In the Alamar Blue assay, a notable approximately 3-fold increase in HUVEC and HDMEC cell viability (as expressed by a relative increase in fluorescence) was observed following incubation with ectosomes derived from WM266-4 and WM1205Lu cells (Figure 7). In addition, ectosomes derived from WM793 cells led to a 1.8-fold increase in HDMEC cell viability. However, when ectosomes were pretreated with antibodies against anti-αvβ5 integrin or cilengitide, the viability of HUVEC and HDMEC cells treated with ectosomes reverted to control values. This suggests that αvβ5 integrin is primarily responsible for the observed increase in endothelial cell viability. Conversely, blocking the function of αvβ3 integrin did not result in significant changes in the effect of ectosomes on target cells.

**Figure 8:**
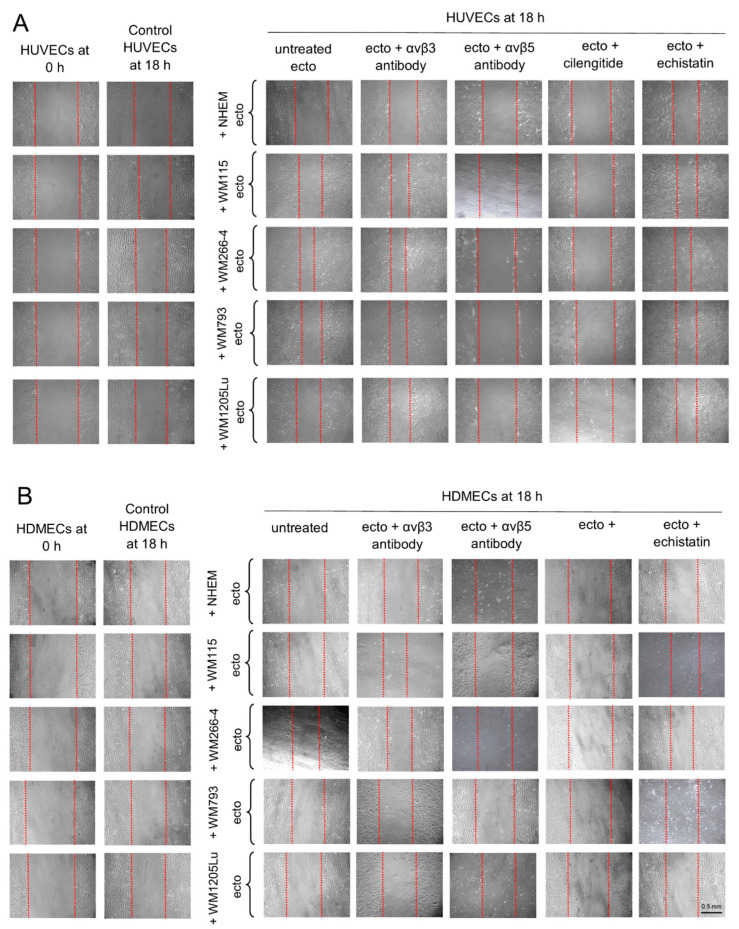
Wound healing assay performed on HUVEC (**A**) and HDMEC (**B**) cells after 18 h of incubation with ectosomes derived from melanocytes and melanoma cells. The isolated ectosomes were pre-incubated with anti-αvβ3 and anti-αvβ5 integrin antibodies or RGD mimetics (cilengitide and echistatin). Wounds (1 mm wide) were created on HUVEC (**A**) and HDMEC (**B**) monolayers and allowed to heal for 18 h without or in the presence of ectosomes. Each wound was photographed immediately after scraping (0 h) and after 18 h. Red dashed lines mark wound borders. Scale bar: 0.5 mm.

**Figure 9:**
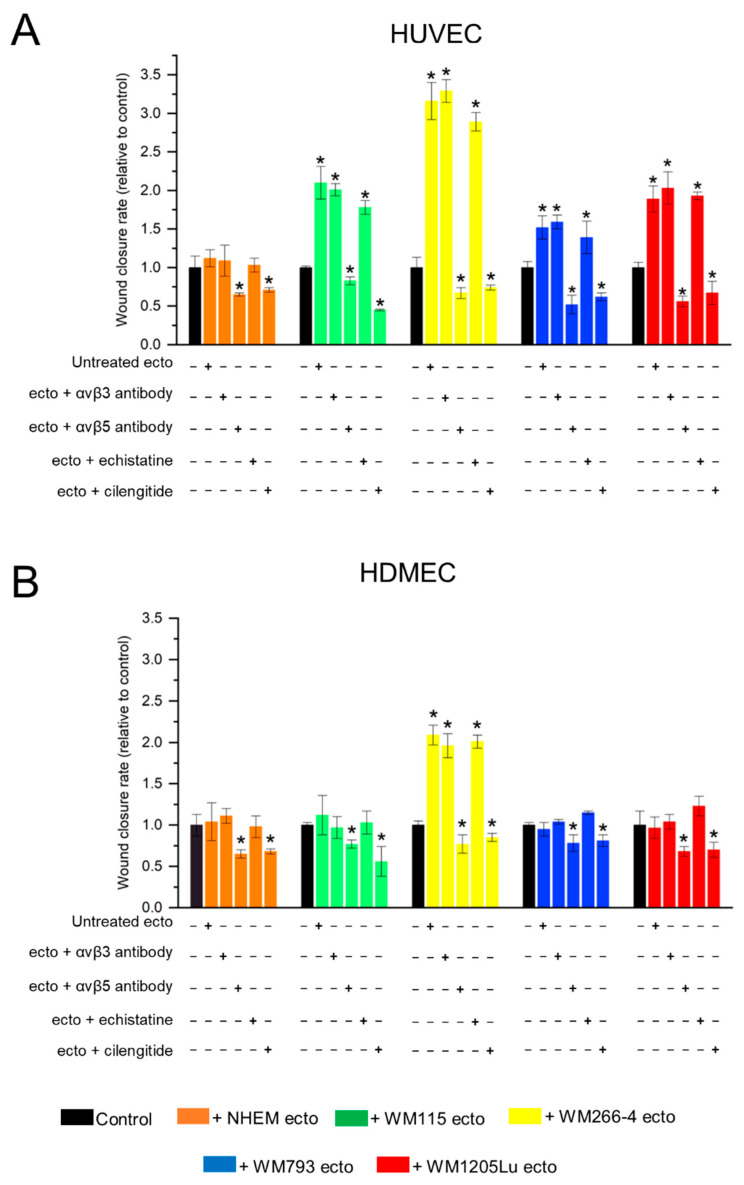
Quantitative analysis of results from wound healing assay carried out on HUVEC (**A**) and HDMEC (**B**) cells after 18 h incubation with melanocyte- and melanoma-derived ectosomes. Isolated ectosomes were pre-incubated with anti-αvβ3 and anti-αvβ5 integrin antibodies or RGD mimetics–cilengitide and echistatin. Wounds (1 mm in width) were created on HUVEC monolayers and allowed to heal for 18 h without or in the presence of ectosomes. Each wound was photographed in 10 separate fields immediately after scraping (0 h) and after 18 h. The average wound closure rate was evaluated by multiple measurements of the wound width on each image. Results were standardized in relation to the untreated control (taken as 1). All experiments were performed in triplicate. “*” indicates statistically significant differences compared to the control (Tukey’s post-hoc test, *p* < 0.05).

Additionally, alterations in the migratory properties of endothelial cells were evaluated using a wound healing assay (Figure 8). Incubation with ectosomes derived from all melanoma cell lines (but not melanocytes) elicited an increase in the rate of wound closure in HUVEC monolayers, ranging from a 1.5-fold increase (for ectosomes derived from WM793) to more than 3-fold increase (for ectosomes derived from WM266-4) (Figure 9A). HDMEC cells exhibited a weaker response, and changes in the degree of wound closure were observed solely after incubation with ectosomes derived from WM266-4 cells (2-fold increase) (Figure 9B). Similar to the viability test, the functional effect exerted by ectosomes was abolished only when ectosomes were pretreated with an antibody against αvβ5 integrin or cilengitide. Moreover, both the anti-integrin αvβ5 antibody and cilengitide reduced the rate of wound closure to values lower than those measured in the control cells.

### 3.6. Functional Impact of Melanoma/Melanocyte-Derived Ectosomes Carrying αvβ3 and αvβ5 Integrins on the Potential for Tube Formation by Endothelial Cells

**Figure 10:**
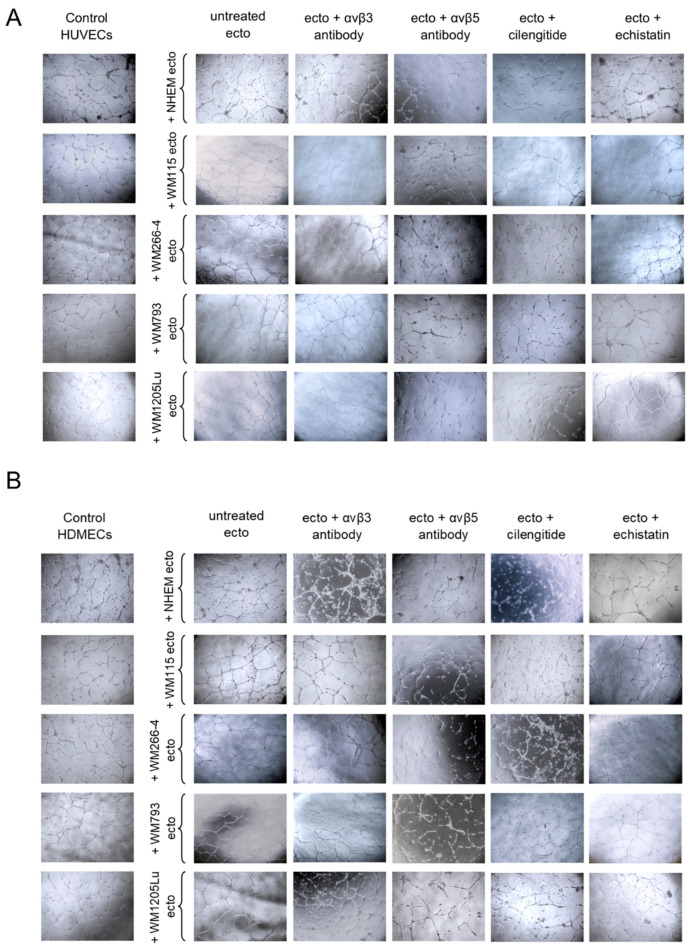
Tube formation assay carried out on HUVEC (**A**) and HDMEC (**B**) cells after 18 h of incubation with melanocyte- and melanoma-derived ectosomes. Isolated ectosomes were pre-incubated with anti-αvβ3 and anti-αvβ5 integrin antibodies or RGD mimetics–cilengitide and echistatin and then added to HUVEC and HDMEC endothelial cells seeded previously on a Geltrex matrix-coated plates. After incubation, HUVEC and HDMEC cell cultures were photographed at 10 separate fields per well. Scale bar: 0.5 mm.

**Figure 11:**
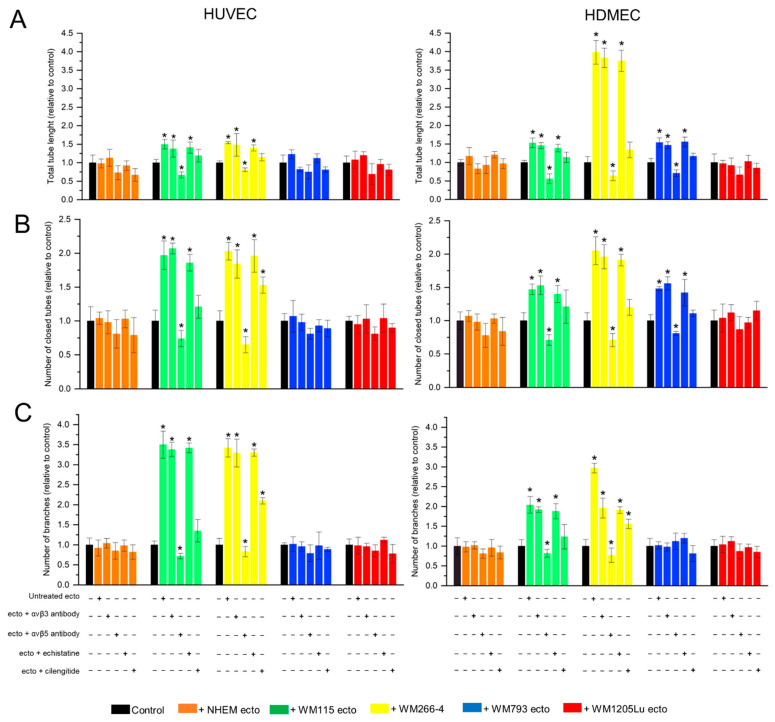
Qualitative analysis of results from tube formation assay carried out on HUVEC and HDMEC cells after 18 h of incubation with melanocyte- and melanoma-derived ectosomes. Isolated ectosomes were pre-incubated with anti-αvβ3 and anti-αvβ5 integrin antibodies or RGD mimetics–cilengitide and echistatin and then added to HUVEC and HDMEC endothelial cells seeded previously on a Geltrex matrix-coated plates. After incubation, HUVEC and HDMEC cell cultures were photographed at 10 separate fields per well. Obtained images were binarized and analyzed in ImageJ with the Angiogenesis Analyzer plug-in. Quantitative image analysis included (**A**) total tube length, (**B**) the number of closed tubes, and (**C**) the number of branches. Results were standardized in relation to the untreated control (taken as 1). All experiments were performed in triplicate. “*” indicates statistically significant differences compared to the control (Tukey’s post-hoc test, *p* < 0.05).

In 2D cell culture, the ability of endothelial cells to form vascular-like structures can be assessed. Culturing cells on a dish coated with a mixture of basement membrane matrix proteins allows endothelial cells to form closed structures resembling cross-sections of blood vessels rather than a compact monolayer. In this study, HUVEC and HDMEC endothelial cells were seeded on a Geltrex matrix-coated plate and then incubated with ectosomes (Figure 10). Incubation with ectosomes derived from WM115 and WM266-4 cells increased tube formation by both endothelial cell lines. Additionally, ectosomes derived from WM793 cells affected selected parameters measured for HDMEC cells (Figure 11). Specifically, for HUVECs, ectosomes derived from WM115 and WM266-4 cells caused approximately a 2-fold increase in the number of closed tubes, a 1.5-fold increase in their total length, and approximately a 3.5-fold increase in the number of branches. The effect of ectosomes derived from WM115 and WM266-4 cells on HDMEC cells was more pronounced. Ectosomes derived from WM115 cells induced a 1.5-fold increase in the number of closed vessels and their length and a 2-fold increase in the number of branches. After incubation of HDMEC cells with ectosomes derived from WM266-4 cells, the number of vessels increased 2-fold, their total length increased 4-fold, and the number of branches increased 3-fold. Moreover, the addition of ectosomes derived from WM793 cells resulted in a 1.5-fold increase in the number of closed vessels and their length in HDMEC cells.

Similar to the viability and migration assays, the utilization of an antibody against αvβ3 integrin or echistatin failed to nullify the effect of ectosomes. However, upon using an antibody against αvβ5 integrin, significant inhibition of tube formation was observed for both endothelial cell lines, with all parameters determined by Angiogenesis Analyzer falling below the control values. Cilengitide also attenuated the proangiogenic effect of ectosomes, although the measured values did not drop below the control levels. Nonetheless, despite the application of cilengitide, ectosomes derived from WM266-4 cells induced a 2- and 1.5-fold increase in the number of branches for HUVECs and HDMECs, respectively. Additionally, in the case of HUVECs, a 1.5-fold increase in the number of closed tubes was observed.

### 3.7. Evaluation of Activation of Proangiogenic Signaling Pathways (αvβ3 Integrin/TNF-α and αvβ5 Integrin/VEGF) in Endothelial Cells after Incubation with Ectosomes

Two cytokine-dependent proangiogenic signaling pathways are recognized: (1) induced by TNF-α and requiring a functional αvβ3 integrin, and (2) induced by VEGF and requiring a functional αvβ5 integrin. In this study, we analyzed changes in gene and protein expression of TNF-α and VEGF in endothelial cells following incubation with integrin-bearing ectosomes.

**Figure 12:**
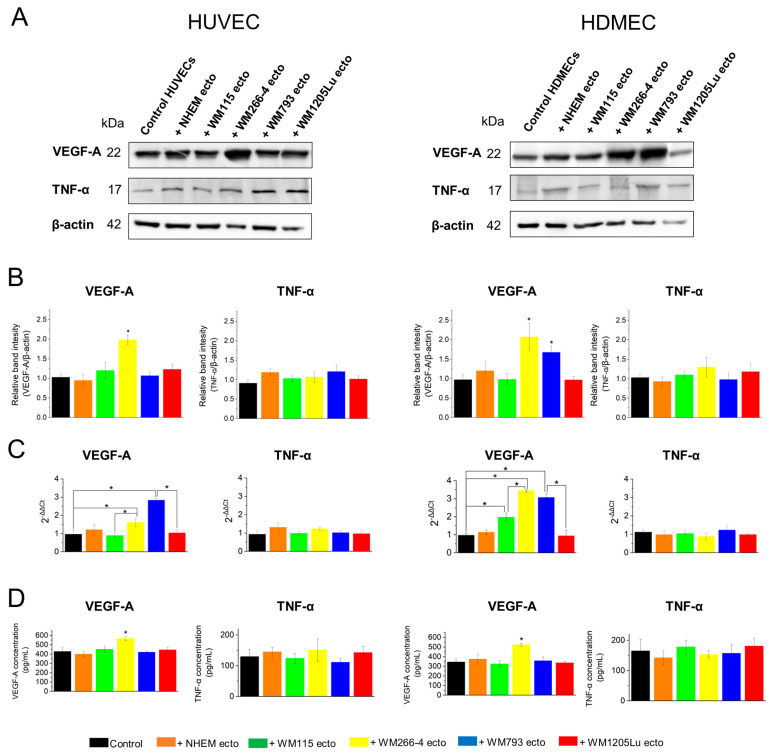
Analysis of changes in gene and protein expression/secretion of TNF-α and VEGF in endothelial cells after 18 h incubation with integrin-bearing ectosomes derived from melanocytes and melanoma cells. (**A**) Representative Western blots from analysis of total VEGF-A and TNF-α protein expression in HUVEC and HDMEC cells after incubation with ectosomes. Thirty micrograms of proteins from whole-cell protein extracts were separated by 10% SDS-PAGE and transferred to the PVDF membrane. Membranes were then probed with rabbit monoclonal primary antibodies, anti-VEGF-A and anti-TNF-α, and goat anti-mouse HRP-conjugated secondary antibodies. Mouse monoclonal anti-β-actin antibody was used as a loading control. (**B**) Densitometric analysis of total VEGF-A and TNF-α protein expression relative to β-actin. (**C**) RT-qPCR analysis of gene expression for *VEGFA* and *TNFA*. Housekeeping (*YWHZ*) and target (*VEGFA*, *TNFA*) gene-specific mRNAs were amplified from 250 ng of cDNA with the use of TaqMan™ Gene Expression Assays. Analysis of relative gene expression was performed using the 2^−ΔΔCt^ method. (**D**) The results of ELISA tests for both proteins were performed on the conditioned medium. All experiments were performed in triplicate. “*” indicates statistically significant differences compared to the control (Tukey’s post-hoc test, *p* < 0.05).

RT-qPCR and immunoblotting showed no changes in the *TNFA* gene and TNF-α protein expression after incubating both endothelial cell lines with ectosomes (Figure 12A–C). However, *VEGFA* gene expression increased in HUVEC cells after incubation with ectosomes from WM266-4 (1.5-fold) and WM793 (2.8-fold) cells (Figure 12C). A more robust response was observed in HDMEC cells, where *VEGFA* gene expression increased upon exposure to ectosomes derived from WM115 (2-fold), WM266-4 (3.5-fold), and WM793 (3-fold) cells (Figure 12C). Interestingly, ectosomes, when compared within isogenic pairs of CM cells, exerted significantly different responses in both HUVEC and HDMEC cells. While ectosomes derived from metastatic WM266-4 cells stimulated *VEGFA* gene expression to a greater extent than ectosomes from their primary isogenic counterpart, i.e., WM115 cells, for ectosomes derived from the second pair (WM793 and WM1205), the effect was more prominent for ectosomes from primary melanoma cells. This suggests a dependence of ectosome function on the genetic background of the cell of their origin.

Concurrent changes in VEGF expression were also noted at the protein level (Figure 12A,B), with immunoblotting showing a 2-fold increase in relative protein expression in both endothelial cell lines after incubation with ectosomes derived from WM266-4 cells. Additionally, ectosomes derived from WM793 cells induced a 1.7-fold increase in VEGF protein expression in HDMEC cells.

Given that TNF-α and VEGF are secreted proteins, ELISA tests for both proteins were conducted on the conditioned medium (Figure 12D). The concentration of TNF-α in culture supernatants from control HUVEC cells was 130 ± 23 pg/mL, and from HDMEC cells was 165 ± 39 pg/mL. No differences in TNF-α secretion were observed for ectosome-treated samples. The concentration of VEGF in the conditioned medium from untreated HUVECs and HDMECs was 427 ± 45 pg/mL and 345 ± 33 pg/mL, respectively (Figure 12D). Incubation of endothelial cells with ectosomes derived from WM266-4 cells increased the concentration of VEGF in the conditioned medium to 567 ± 21 pg/mL (HUVEC) and 523 ± 16 pg/mL (HDMEC).

## 4. Discussion

### 4.1. The Role and Expression of αvβ3 and αvβ5 Integrins in Melanoma Cells and Melanoma-Derived Ectosomes

In recent years, evidence has emerged that various integrin receptors can be transferred by EVs between cancer cells and from cancer cells to non-transformed cells within the tumor microenvironment or metastatic niches. Moreover, the EV-mediated transfer of integrins can lead to recipient cells acquiring specific phenotypes or functions [11,12,13]. Transferred integrin receptors have been implicated in determining the organotropism of metastasizing cells [26] and contributing to resistance to certain therapeutic agents [27]. Several integrins, notably αvβ3 and αvβ5 integrins, possess well-documented proangiogenic potential [28]. They modulate the adhesion of (micro)vascular endothelial cells to ECM proteins and initiate proangiogenic intracellular signaling. However, whether the proangiogenic effect exerted by EVs on recipient cells is mediated in any way by integrins present in vesicular cargo remains an open question.

So far, ectosomes released by only a few types of cancer have been evaluated as proangiogenic factors, and none of the studies have been concerned with melanoma [29]. In melanoma, an increased expression of αvβ3 integrin correlates with conversion from the radial to the vertical growth phase [30]; however, there are metastatic melanoma cell lines with low or no αvβ3 integrin expression [31]. The expression of the β3 integrin subunit alone has been shown to correlate positively with lung metastasis and worse survival rates [23]. Interestingly, studies on various cell lines, mice, and human specimens have shown that αvβ5 integrin expression is lost in advanced stages of melanoma, while αvβ3 integrin expression is acquired during the vertical growth phase [24,25]. Such a phenomenon has direct functional consequences for melanoma cells in terms of their adhesive properties and potential angiogenesis.

In the present study, we analyzed the total protein expression of both receptors in four melanoma cell lines using immunoblotting (Figure 5), which yielded results consistent with previous findings. Metastatic CM cell lines exhibited higher expression of αvβ3 integrin compared to their primary isogenic counterparts. Regarding αvβ5 integrin expression, no significant difference was observed between WM115 and WM266-4 CM cell lines. However, metastatic WM1205Lu cells displayed lower expression of this receptor compared to the primary WM793 cell line. This pattern aligns with the observed loss of αvβ5 integrin often noted in advanced stages of melanoma [24,25].

Moreover, considering that the specific cargo of EVs is determined by the molecular content of the parental cell, we anticipated that changes in αvβ3 integrin and αvβ5 integrin expression would be reflected in the molecular cargo of EVs. Through immunoblotting, we demonstrated that αvβ3 integrin expression was consistent across melanoma cells and their derivative ectosome samples. Given that the process of protein sorting into ectosomes is selective and involves a limited number of membrane and cytoplasmic proteins, it is plausible that the proportional abundance of αvβ3 integrin in the ectosomal cargo is higher than in the parental cells. Regarding αvβ5 integrin, similar trends were observed, except in the case of ectosomes derived from WM793 cells. Here, higher expression of αvβ5 integrin was noted in ectosomes derived from WM793 cells compared to the cells releasing them. This observation may suggest preferential incorporation of this receptor into ectosomes at early stages of melanoma, potentially elucidating the diminished levels of αvβ5 integrin during disease progression.

### 4.2. Transfer of αvβ3 and αvβ5 Integrins via Melanoma-Derived Ectosomes Is Associated with Alterations in αvβ3/αvβ5 Integrin Protein and Gene Expression in Recipient Endothelial Cells

The predominant focus of research on integrins in angiogenesis has centered on αvβ3 integrin, a vitronectin receptor crucial for angiogenesis and upregulated in developing blood vessels [32]. This study aimed to investigate whether the transfer of tumor-derived αvβ3 integrin to endothelial cells via ectosomes contributes to the overall angiogenic potential of melanoma cells. Previous studies have demonstrated that exosomes derived from PC3 and CWR22Pc prostate cancer cells bearing αvβ3 integrin enhanced the adhesion and migration of non-tumorigenic BHP-1 cells [13]. Additionally, exosomes from the plasma of prostate cancer patients transferred αvβ3 integrin to β3 integrin-negative recipient cells, imparting β3 integrin-related ligand binding activity [33].

Moreover, recent research has shown that blocking of αvβ3 integrin present on the EV surface impedes their binding and uptake by recipient cells [34], indicating the direct involvement of αvβ3 integrin in EV–recipient cell interactions. In this study, while the impact of αvβ3 and αvβ5 integrins on ectosome uptake by endothelial cells was not analyzed, the successful incorporation of fluorescent-stained ectosomes by HUVEC and HDMEC was demonstrated using three independent methods (Figure 3). Regardless of the ectosome origin, endothelial cells efficiently internalized the ectosomes, whereas quantitative differences in fluorescence intensity were more pronounced between different endothelial cell lines treated with ectosomes of the same origin rather than within a single endothelial cell line treated with ectosomes of different origins. This suggests that recipient cell-specific characteristics may predominantly dictate the interactions between ectosome and endothelial cells rather than the molecular cargo within ectosomes.

Studies on exosomes derived from prostate cancer cells have demonstrated that incubation with exosomes leads to an increase in αvβ3 integrin protein expression in recipient cells [13,33,34]. We similarly observed an elevation in total αvβ3 integrin protein expression in both HUVECs and HDMECs following incubation with ectosomes from WM1205Lu cells (Figure 5). This increase may be attributed to the higher αvβ3 integrin expression observed in these specific ectosomes. Moreover, the most notable rise in αvβ5 integrin expression in HUVECs and HDMECs was observed after incubation with ectosomes derived from WM793 cells, correlating with the highest expression of αvβ5 integrin in these ectosomes. These findings indicate a correlation between the relative content of both integrin receptors in ectosomes and the subsequent increase in their expression in recipient endothelial cells. 

Additionally, we examined changes in the surface expression of αvβ3 and αvβ5 integrins via flow cytometry (Figure 6), although these alterations were less pronounced compared to those observed using immunoblotting. This disparity likely stems from the internalization of integrin-bearing ectosomes by recipient cells rather than direct incorporation into the outer cell membrane. The slight decreases in integrin surface expression observed for HUVECs under WM793- and WM1205Lu-ectosome treatment may be linked to subtle rearrangements within the outer cell membrane, potentially occurring during ectosome endocytosis.

The transfer of integrin molecules to endothelial cells likely occurs primarily through the delivery of proteins via ectosomes, although the possibility of ectosomes inducing de novo synthesis of integrin subunits by stimulating gene expression cannot be entirely ruled out. In our RT-qPCR analysis, we found no significant changes in gene expression for integrin subunits in HDMEC cells. Conversely, in HUVEC cells, we observed an upregulation in the expression of β3 and β5 integrin subunit genes (*ITGB3* and *ITGB5*) following incubation with ectosomes, while the gene expression of αv integrin subunit remained unchanged. These findings suggest that ectosomes have the capacity to stimulate gene expression for integrin subunits and promote their de novo synthesis in HUVECs, alongside facilitating the transfer of functional receptors from melanoma cells. In contrast, we did not observe any genomic impact of ectosomes on HDMEC cells, indicating that the effect mediated by the same ectosomes is strongly dependent on the type of recipient cell.

### 4.3. Melanoma-Derived Ectosomes Bearing αvβ3 and αvβ5 Integrins Exert Proangiogenic Effect on Recipient Endothelial Cells

Recent studies have demonstrated that the absence of αvβ3 integrin in melanoma tumor cells downregulates the angiogenic switch, leading to reduced tumor growth and microvessel density in mice [12]. In the same study, experiments utilizing conditioned media from melanoma cells, with or without αvβ3 integrin expression, revealed a significant decrease in the proliferation rate of HUVEC cells when deprived of αvβ3 integrin, suggesting a functional impact of αvβ3 integrin present in melanoma secretome on endothelial cells. However, given the use of unfractionated conditioned media, further investigations were needed to verify the specific components responsible for these effects.

In this study, we aimed to elucidate the effect of isolated ectosomes derived from melanoma/melanocytes on the viability and migratory capacity of recipient HUVEC and HDMEC cells. To determine the contribution of αvβ3 and αvβ5 integrins to the observed proangiogenic effects, we employed specific antibodies and RGD mimetics, i.e., echistatin (αvβ3 integrin inhibitor) and cilengitide (an inhibitor of both αvβ3 and αvβ5 integrin). In the Alamar Blue assay (Figure 7), we observed enhanced viability of both endothelial cell lines after incubation with ectosomes derived from metastatic CM cell lines. Subsequent wound healing assay (Figure 8 and Figure 9) revealed an acceleration in the rate of wound closure in HUVEC monolayers upon incubation with ectosomes derived from all CM cell lines, with the exception of melanocytes. HDMEC cells exhibited a milder response, with noticeable changes in wound closure observed only upon treatment with ectosomes derived from the WM266-4 CM cells. Crucially, the proangiogenic effects mediated by ectosomes were effectively abrogated upon pretreatment with an antibody against αvβ5 integrin or cilengitide. This underscores the pivotal role of ectosomal αvβ5 integrin in promoting proliferation and migration of recipient endothelial cells while blocking the function of ectosomal αvβ3 integrin did not impact these properties.

Furthermore, we performed a 2D tube formation assay (Figure 10 and Figure 11). Incubation with ectosomes derived from WM115 and WM266-4 cells significantly increased tube formation by both endothelial cell lines, as evidenced by enhancements in total tube length, number of closed tubes, and number of branches. Notably, ectosomes derived from WM793 cells also impacted selected parameters measured for HDMEC cells. Consistent with the findings from the Alamar Blue and wound healing assays, the use of anti-αvβ3 integrin antibody or echistatin did not mitigate the proangiogenic effect of ectosomes. However, when anti-αvβ5 antibody or cilengitide was employed, significant inhibition of tube formation was observed for both endothelial cell lines, resulting in a decrease in all assayed parameters.

Results from functional studies can be explained by the existence of two distinct cytokine-dependent angiogenic signaling pathways: one induced by TNF-α and requiring functional αvβ3 integrin, and the other induced by VEGF and dependent on functional αvβ5 integrin [14]. To date, no studies have explored which of the aforementioned mechanisms predominates for EV-associated integrins, nor have they investigated whether the frequency of the αvβ3 integrin/TNF-α or αvβ5 integrin/VEGF-dependent pathway changes with tumor progression. In this study, no changes in the *TNFA* gene and TNF-α protein expression after incubation with ectosomes were observed (Figure 12). However, *VEGFA* gene expression increased in both endothelial cell lines following incubation with ectosomes derived from WM793- and WM266-4 cells. Moreover, changes in VEGF expression were also observed at the protein level in both endothelial lines after exposure to ectosomes derived from the WM266-4 cells. Additionally, ELISA assays showed no differences in TNF-α secretion for ectosome-treated samples, while the concentration of VEGF in the conditioned medium from HUVEC and HDMEC cells increased after incubation with ectosomes derived from the WM266-4 cells. These findings suggest that ectosomes derived from melanoma cells, despite inducing changes in the surface and total αvβ3 integrin expression, do not activate the αvβ3 integrin/TNF-α pathway in recipient endothelial cells. However, they do activate the αvβ5 integrin/VEGF pathway.

Finally, targeting αvβ3 integrin function with specific antibodies or low-molecular-weight antagonists, such as RGD mimetics, has been shown to inhibit angiogenesis in various in vivo models, including mouse melanoma [12]. However, despite promising preclinical results, clinical trials using agents like the monoclonal antibody Vitaxin [35] and the RGD mimetic cilengitide [36] have not met expectations in terms of inhibiting tumor angiogenesis. While cilengitide showed some effectiveness in phase II clinical trials for glioblastoma multiforme, it proved ineffective in phase III trials [37].

While cilengitide showed minimal clinical efficacy as a single-agent therapy in melanoma [38], it has been demonstrated to reduce the invasiveness and vasculogenic mimicry of neuropilin-1(NRP-1)-expressing melanoma cells by inhibiting αvβ5 integrin and αvβ5 integrin/NRP-1/VEGF-A signaling [39]. Similarly, the results of the present study suggest that targeting αvβ5 integrin may be more effective in inhibiting angiogenesis than targeting αvβ3 integrin. Therefore, given the significant contribution of EVs to the intercellular transfer of integrins, further studies are needed to elucidate the intricate effects of integrins in the tumor microenvironment, with particular attention to the role of EV-mediated transfer mechanisms.

## 5. Conclusions

The study presented here was designed to investigate whether and to what extent ectosomes, as part of the melanoma secretome, mediate the proangiogenic effects of tumor-derived αvβ3 and αvβ5 integrins. It represents the first investigation into the interactions between melanoma-derived ectosomes and endothelial cells, as well as the role of αvβ3 and αvβ5 integrins present in this understudied EV population in tumor angiogenesis. The findings of this study reveal that melanoma-derived ectosomes induce changes in the expression of αvβ3 and αvβ5 integrins in recipient endothelial cells, leading to increased viability, migratory properties, and tube formation potential. However, the extent of proangiogenic stimulation varies depending on the types of cells releasing ectosomes (from different stages of melanoma) and the recipient cells (HUVECs vs. HDMECs). Furthermore, the use of anti-integrin antibodies and integrin-blocking peptides suggests a more prominent role for the αvβ5 integrin/VEGF pathway compared to αvβ3integrin/TNF-α signaling in the interactions between ectosomes and endothelial cells studied.

In conclusion, these findings contribute to a deeper understanding of the mechanisms underlying pathological angiogenesis, particularly in the context of melanoma. This understanding is crucial for the development of more effective anti-angiogenic strategies for melanoma treatment, an area where current therapies are limited. Moreover, the identification of various proangiogenic factors in EVs, beyond classical factors such as VEGF, IL-6 or MMPs, underscores the importance of exploring different EV populations to fully exploit their potential in antiangiogenic therapy.

## Figures and Tables

**Table 1:** Summary of housekeeping and target genes TaqMan™ probes set.

Symbol	Gene Name (Assay ID, TaqMan Probes; Amplicon Size bp)	Location (GeneCards)	Description
**Housekeeping gene:**			
*YWHAZ*	tyrosine 3-monooxygenase/tryptophan 5-monooxygenase activation protein zeta (Hs01122444_g; 120 bp)	8q22.3	Mediator of various signaling pathways
**Target genes:**			
*ITGAV*	integrin subunit alpha V (Hs00233808_m1; 64 bp)	2q32.1	Cell adhesion receptor
*ITGB3*	integrin subunit beta 3 (Hs01001469_m1; 59 bp)	7q21.32	Cell adhesion receptor
*ITGB5*	integrin subunit beta 5 (Hs00174435_m1; 78 bp)	3q21.2	Cell adhesion receptor
*VEGFA*	vascular endothelial growth factor A (Hs00900055_m1; 59 bp)	6p21.1	Angiogenesis inducer
*TNFA*	tumor necrosis factor-alpha (Hs00174128_m1; 80 bp)	6p21.33	Proinflammatory cytokine

## Data Availability

The original contributions presented in the study are included in the article/Supplementary Material; further inquiries can be directed to the corresponding author.

## Supplementary Materials

The following supporting information can be downloaded at: https://www.mdpi.com/article/10.3390/cells13161336/s1, Figure S1. Flow cytometry analysis of αvβ3 integrin surface expression in HDMEC cells following 18 h of incubation with melanocyte- and melanoma-derived ectosomes, Figure S2. Flow cytometry analysis of αvβ5 integrin surface expression in HDMEC cells following 18 h of incubation with melanocyte- and melanoma-derived ectosomes.
