# Supplementary material for: The Proangiogenic Effects of Melanoma-Derived Ectosomes Are Mediated by αvβ5 Integrin Rather than αvβ3 Integrin
Source: Cells. 2024 Aug 12;13(16):1336. doi: 10.3390/cells13161336 (PMC11352487; doi:10.3390/cells13161336)
Supplement: Supplementary file 1 [file cells-13-01336-s001.zip › cells-3104552-supplementary.pdf]

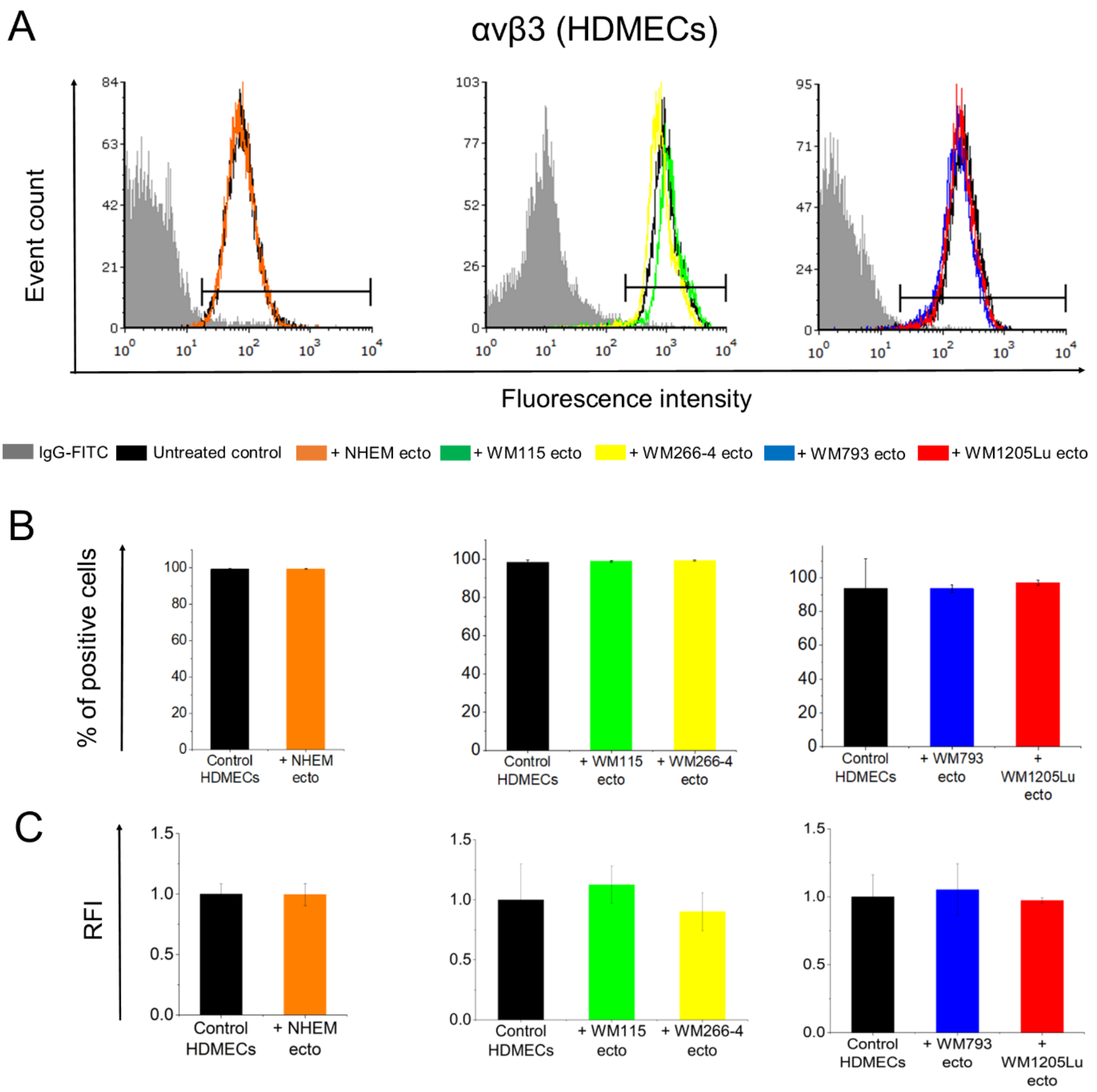

Figure S1. Flow cytometry analysis of αvβ3 integrin surface expression in HDMEC cells after 18-hour incubation with melanocyte- and melanoma-derived ectosomes. After incubation, 5×10^4 cells were collected, indirectly labelled with rabbit monoclonal primary anti-αvβ3 antibody and secondary FITC-conjugated goat anti-rabbit IgG, and then analyzed by flow cytometry. A – representative histograms. The gray-shaded histograms depict background staining acquired from secondary staining controls. The histogram markers were set based on the signal derived from these controls, designating the histogram sections that correspond to positive HUVEC cells. B – surface expression of αvβ3 integrin on HUVEC cells presented as the percentage of positive cells and C – relative fluorescence intensity of specific staining. All experiments were performed in triplicate. "*" indicates statistically significant differences compared to control (Tukey's post hoc test, $p < 0.05$).

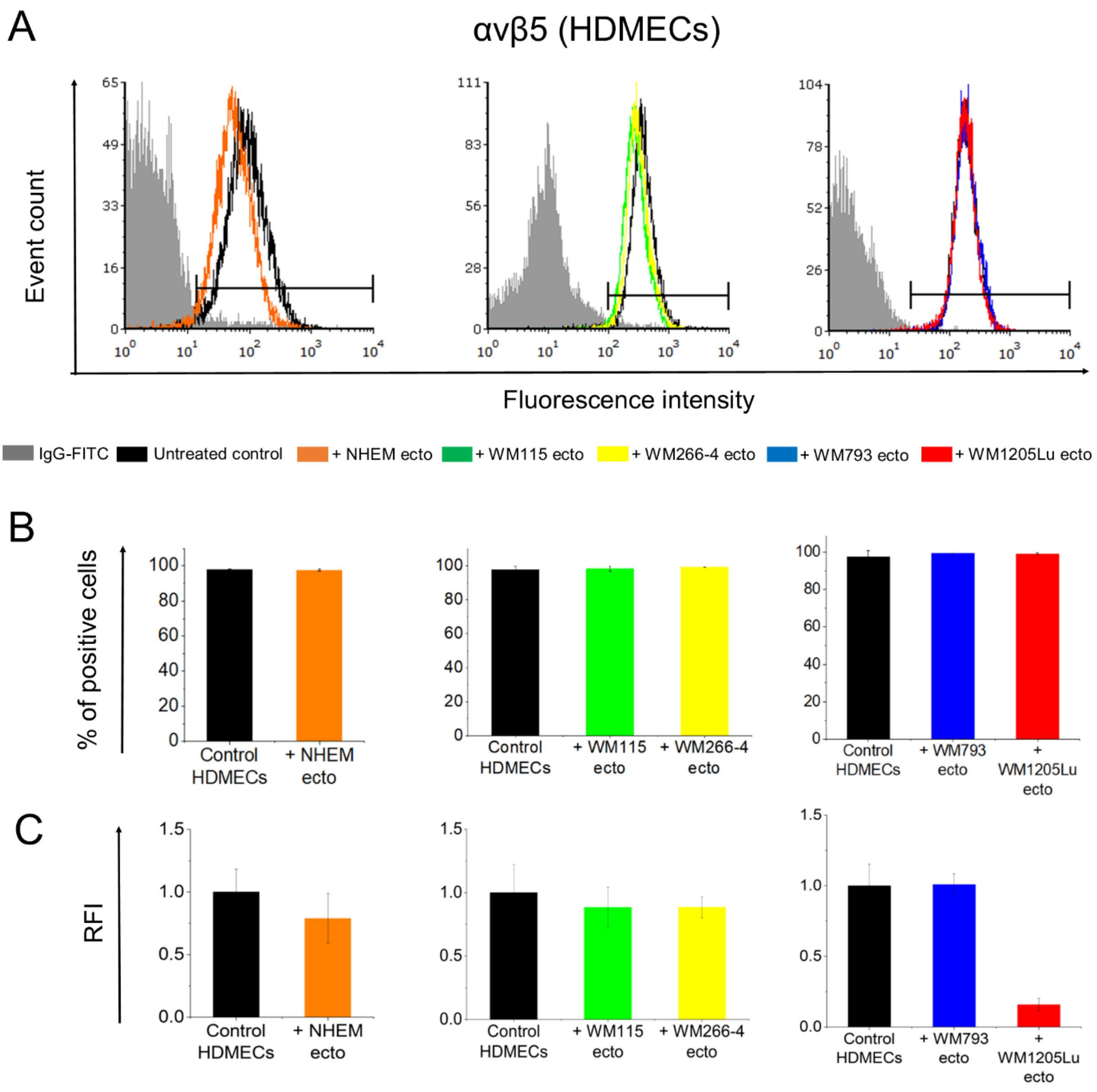

Figure S2. Flow cytometry analysis of αvβ5 integrin surface expression in HDMEC cells after 18-hour incubation with melanocyte- and melanoma-derived ectosomes. After incubation, 5×10^4 cells were collected, indirectly labelled with rabbit monoclonal primary anti-αvβ3 antibody and secondary FITC-conjugated goat anti-rabbit IgG, and then analyzed by flow cytometry. A – representative histograms. The gray-shaded histograms depict background staining acquired from secondary staining controls. The histogram markers were set based on the signal derived from these controls, designating the histogram sections that correspond to positive HUVEC cells. B – surface expression of αvβ3 integrin on HUVEC cells presented as the percentage of positive cells and C – relative fluorescence intensity of specific staining. All experiments were performed in triplicate. “*” indicates statistically significant differences compared to control (Tukey’s post hoc test, $p < 0.05$).
